# Supplementary dydrogesterone is beneficial as luteal phase support in artificial frozen-thawed embryo transfer cycles compared to micronized progesterone alone

**DOI:** 10.3389/fendo.2023.1128564

**Published:** 2023-03-13

**Authors:** Angela Vidal, Carolin Dhakal, Nathalie Werth, Jürgen Michael Weiss, Dirk Lehnick, Alexandra Sabrina Kohl Schwartz

**Affiliations:** ^1^ Division of Reproductive Medicine and Gynecological Endocrinology, Lucerne Cantonal Hospital, Lucerne, Switzerland; ^2^ Fertisuisse Center for Reproductive Medicine, Olten, Switzerland; ^3^ Kinderwunschteam, Berlin, Berlin, Germany; ^4^ Biostatistics and Methodology CTU-CS (Clinical Trial Unit – Central Switzerland), University of Lucerne, Lucerne, Switzerland; ^5^ Department of Obstetrics and Gynecology, Bern University Hospital, Inselspital, University of Bern, Bern, Switzerland

**Keywords:** dydrogesterone, micronized progesterone gel, luteal phase support, frozen embryo transfer, clinical pregnancy rate, live birth rate

## Abstract

**Introduction:**

The number of frozen embryo transfers increased substantially in recent years. To increase the chances of implantation, endometrial receptivity and embryo competency must be synchronized. Maturation of the endometrium is facilitated by sequential administration of estrogens, followed by administration of progesterone prior to embryo transfer. The use of progesterone is crucial for pregnancy outcomes. This study compares the reproductive outcomes and tolerability of five different regimens of hormonal luteal phase support in artificial frozen embryo transfer cycles, with the objective of determining the best progesterone luteal phase support in this context.

**Design:**

This is a single-center retrospective cohort study of all women undergoing frozen embryo transfers between 2013 and 2019. After sufficient endometrial thickness was achieved by estradiol, luteal phase support was initiated. The following five different progesterone applications were compared: 1) oral dydrogesterone (30 mg/day), 2) vaginal micronized progesterone gel (90 mg/day), 3) dydrogesterone (20 mg/day) plus micronized progesterone gel (90 mg/day) (dydrogesterone + micronized progesterone gel), 4) micronized progesterone capsules (600 mg/day), and (5) subcutaneous injection of progesterone 25 mg/day (subcutan-P4). The vaginal micronized progesterone gel application served as the reference group. Ultrasound was performed after 12-15 days of oral estrogen (≥4 mg/day) administration. If the endometrial thickness was ≥7 mm, luteal phase support was started, up to six days before frozen embryo transfer, depending on the development of the frozen embryo. The primary outcome was the clinical pregnancy rate. Secondary outcomes included live birth rate, ongoing pregnancy, and miscarriage and biochemical pregnancy rate.

**Results:**

In total, 391 cycles were included in the study (median age of study participants 35 years; IQR 32-38 years, range 26–46 years). The proportions of blastocysts and single transferred embryos were lower in the micronized progesterone gel group. Differences among the five groups in other baseline characteristics were not significant. Multiple logistic regression analysis, adjusting for pre-defined covariates, showed that the clinical pregnancy rates were higher in the oral dydrogesterone only group (OR = 2.87, 95% CI 1.38–6.00, p=0.005) and in the dydrogesterone + micronized progesterone gel group (OR = 5.19, 95% CI 1.76–15.36, p = 0.003) compared to micronized progesterone gel alone. The live birth rate was higher in the oral dydrogesterone-only group (OR = 2.58; 95% CI 1.11–6.00; p=0.028) and showed no difference in the smaller dydrogesterone + micronized progesterone gel group (OR = 2.49; 95% CI 0.74–8.38; p=0.14) compared with the reference group.

**Conclusion:**

The application of dydrogesterone in addition to micronized progesterone gel was associated with higher clinical pregnancy rate and live birth rate and then the use of micronized progesterone gel alone. DYD should be evaluated as a promising LPS option in FET Cycles.

## Introduction

Assisted reproductive technology (ART) has progressed steadily in recent years, worldwide. Frozen-thawed embryo transfer cycles (FETs) have been widely applied globally since 1983. Their use has increased more rapidly during the last decade due to improved laboratory techniques, e.g., embryo cryopreservation or vitrification (1–4). In Switzerland, the reasons for performing FET include the prevention of ovarian hyperstimulation syndrome, the synchronicity of endometrial maturation, pre-implantation genetic testing, and fertility preservation for medical reasons. In addition, FET is associated with improved endometrial thickness, and improved endometrial receptivity, as well as more convenient planning (5–7). The success of a FET cycle relies on the synchronization of endometrial growth, embryo maturation, and timely endometrial secretory transformation induced by progesterone (P4) (8, 9).

The two main types of endometrial preparation are (1) the natural cycle FET (NC-FET), which is based on physiological estrogen production through follicular growth, ovulation induction, and consequently the formation of the corpus luteum producing progesterone (10). NC-FET is preferred because of the positive effect on the production of corpus luteum. In contrast, the hormone replacement therapy (HRT) and frozen embryo transfer (HRT-FET) cycle uses either constant or increasing sequential doses of exogenous estrogens to stimulate endometrial growth and inhibit follicular maturation. To transform the endometrium, exogenous P4 is applied before the embryo transfer (6, 9, 10). The role of progesterone and endometrium stability during FET is essential to support implantation and maintain pregnancy (10, 11). In HRT-FET cycles, adequate luteal phase support (LPS) is essential, given the absence of endogenous progesterone production due to the lack of a corpus luteum (12).

Currently, the best administration option of progesterone in terms of efficacy in FET-HRT is not known, since most studies on progesterone are based on fresh *in vitro* fertilization (IVF) cycles. Tolerability and safety profiles vary, depending on the route of the administration (13). There are four routes of administration of P4 in LPS, including oral, intravaginal, subcutaneous, and rectal or intramuscular (8).

In recent years, oral dydrogesterone (DYD) took on an important role in LPS. DYD has high oral bioavailability, high specificity for P4 receptors, and a good tolerability profile (13). In 2017 and 2018, the Lotus I and II studies were published, which demonstrated that oral DYD is superior to micronized progesterone capsules (MPC) or micronized progesterone gel (MPG) for LPS in fresh IVF cycles (14, 15) only. The aim of this study was to compare five different P4 regimens (DYD, MPG, DYD+MPG, MPC, and subcutan-P4) in the HRT-FET cycles of a real-world population of women undergoing ART.

## Materials and methods

### Study population and selection criteria

This single-center retrospective observational study was conducted between 2013 and 2019 in the Division of Reproductive Medicine and Gynecological Endocrinology, Lucerne Cantonal Hospital, Switzerland. The study included 391 cycles of women aged 18–46 years who underwent FET, with an endometrial thickness of ≥7 mm on the secretory transformation day. The exclusion criteria were intracytoplasmic sperm injection (ICSI), fertilization with testicular sperm extraction sperm, treated congenital uterine malformations, and uterine fibroids.

### Data collection

All data were collected in a medical database. Informed consent was signed by all patients prior to ART procedures.

### Intervention: HRT-FET protocol

To prepare the endometrium for FET, all women started taking 2 mg oral estradiol valerate (Progynova^®^, Bayer, Germany) on the first day of the cycle, increasing gradually from 2 mg per day up to a maximum of 8 mg/day. After 12-16 days of oral estrogenic preparation, transvaginal ultrasound was conducted to assess endometrial thickness. The cycle was canceled if the endometrial thickness was <7 mm after the 16th day of treatment with the described HRT. LPS was initiated if there was an adequate endometrial growth of ≥ 7 mm. The estradiol valerate dosage was continued at least for 14 days until the β-hCG measurement.

From 2013 to 2017, the most common treatment with progesterone was MPG, although also MPC und subcutan-4 were part of LPS in this period. As of 2018, treatment was administered with DYD and the combination of DYD+MPG. The LPS was administered according to the patient’s preference.

The patients were divided into five groups based on different progesterone applications, as follows (1): oral DYD 30 mg/day (Duphaston^®^ Mylan Pharma GmbH) (2), vaginal progesterone gel 90 mg/day (MPG; Crinone^®^ Merck Serono, Switzerland) (3), a combination of DYD 20 mg/day and MPG 90 mg/day (DYD+MPG) (4), MPC 600 mg (Utrogestan^®^ Vifor SA), and (5) subcutaneous injection of 25 mg/day progesterone (subcutan-P4) (Prolutex^®^ Institut Biochimique SA IBSA).

Embryo quality was assessed on the third and fifth days (16) and transfers were performed on either day three or day five, depending on the quality and number of embryos and on clinical indications. A maximum of two embryos were transferred. Cleavage stage embryos were transferred on day four after initiation of luteal support, and blastocyst transfers were conducted on the sixth day of luteal support. In the event of pregnancy, the administration of progesterone was continued until 12 weeks gestation.

### Outcome measures

The primary outcome was the clinical pregnancy rate (CPR) (fetal heartbeat in ultrasound). The secondary outcomes were live birth rate (LBR) per embryo transfer (16), biochemical pregnancy (detection of β-hCG in serum or urine) rate, miscarriage rate (pregnancy loss at < 12 weeks), and pregnancy outcomes, including preeclampsia, vaginal bleeding, and gestational diabetes.

### Statistical analyses

MPG was chosen as the reference group due to the higher number of cycles and was then compared to the different types of progesterone (DYD, DYD+MPG, MPC, and subcutan-4). Categorical data are expressed as absolute and relative frequencies (**Table 1**). Continuous variables are presented using descriptive statistics, including median, first and third quartile (Q1, Q3), and range. A multiple logistic regression analysis was conducted, with adjustment for pre-defined potential covariates (age group, type of sterility, stage (cleavage vs. blastocyst) combined with the number of transferred embryos) to calculate odds ratios (ORs) and corresponding 95% confidence intervals (CIs) for CPR and LBR (**Table 2**). For comparison, the unadjusted ORs were also reported. The level of significance was set to 5% (two-sided). Due to the exploratory nature of the study, no adjustments for multiplicity were applied.

**Table 1:** Demographic characteristics of the study population.

	MPG	DYD	DYD + MPG	MPC	Subcutan-P4	P-value
	(N = 281)	(N = 52)	(N = 17)	(N = 37)	(N = 4)	
Age *	35 (28-32)	33 (31-35.5)	33 (28-36)	35 (34-40)	37 (34-40.5)	P = 0.003^a^
Typ of Sterility						
Primary	252 (89.7%)	46 (88.5%)	16 (94.1%)	30 (81.1%)	2 (50.0%)	P = 0.096^b^
Secundary	29 (10.3%)	6 (11.5%)	1 (5.9%)	7 (18.9%)	2 (50.0%)	
Fertilisation						
IVF	123 (43.8%)	23 (44.2%)	8 (47.1%)	13 (35.1%)	0	P = 0.43^b^
ICSI	158 (56.2%)	29 (55.8%)	9 (52.9%)	24 (64.9%)	4 (100.0%)	
Previous transfers						
0	9 (3.2%)	0	1 (5.9%)	4 (10.8%)	0	P = 0.095^b^
1	75 (26.7%)	16 (30.8%)	1 (5.9%)	9 (24.3%)	0	
>2	197 (70.1%)	36 (69.2%)	15 (88.2%)	24 (64.9%)	4 (100.0%)	
Reason infertility						
Tubal factor	14 (5.0%)	2 (3.8%)	0	2 (5.4%)	2 (50.0%)	P < 0.001^b^
Cryptozoospermia	6 (2.1%)	1 (1.9%)	2 (11.8%)	5 (13.5%)	0	
Oligoasthenoteratozoospermia	14 (5.0%)	2 (3.8%)	5 (29.4%)	3 (8.1%)	0	
Endometriosis III-IV°	7 (2.5%)	0	0	0	0	
Endometriosis I-II°	15 (5.3%)	4 (7.7%)	0	2 (2.7%)	0	
Anovulation/dysovulation/PCOS	199 (70.8%)	33 (63.5%)	10 (58.8%)	17 (45.9%)	2 (50.0%)	
idiopathic	26 (9.3%)	10 (19.2%)	0	9 (24.3%)	0	
Embryo transfer						
blastocyst stage	56 (19.9%)	41 (78.8%)	14 (82.4%)	32 (86.5%)	4 (100.0%)	P < 0.001^b^
cleavage stage	225 (80.1%)	11 (21.2%)	3 (17.6%)	5 (13.5%)	0	
Number of transferred embryos						
1	98 (34.9%)	44 (84.6%)	12 (70.6%)	33 (89.2%)	3 (75.0%)	P < 0.001^b^
2	183 (65.1%)	8 (15.4%)	5 (29.4%)	4 (10.8%)	1 (25.0%)	

Values are presented as numbers or percentages (%). Age represents the first and third quartile percentiles. * Median (Q1-Q3), ^a^ Kruskal-Wallis test, ^b^ Fisher’s exact test. Oral Dydrogesterone 30 mg/day (DYD), vaginal progesterone gel 90 mg/day (MPG), a combination of DYD 20 mg/day and MPG 90 mg/day (DYD+MPG), vaginal progesterone 600 mg ((MPC) and subcutaneous injection of 25 mg/day progesterone (subcutan-P4).

**Table 2:** Pregnancy outcomes.

Pregnancy Outcomes						
	MPG	DYD	DYD + MPG	MPC	Subcutan-P4	P-Value
	(N = 281)	(N = 52)	(N = 17)	(N = 37)	(N = 4)	
Clinical pregnancy	50 (17.8%)	22 (42.3%)	10 (58.8%)	3 (8.1%)	1(25.0%)	P < 0.001^a^
Ongoing pregnancy	33 (11.7%)	14 (26.9%)	5 (29.4%)	2 (5.4%)	1(25.0%)	P = 0.005^a^
Live births	33 (11.7%)	14 (26.9%)	5 (29.4%)	2 (5.4%)	1(25.0%)	P = 0.005^a^

^a^ Fisher’s exact test.

All statistical analyses were conducted with STATA (Version 16.1 or higher, StataCorp, College Station, Texas, USA).

### Ethical approval

The study was approved by the local ethics committee and was registered at the Business Administration System for Ethics Committees (BASEC 2020-01527). It was conducted in accordance with the Declaration of Helsinki.

## Results

### Study population

**Figure 1:**
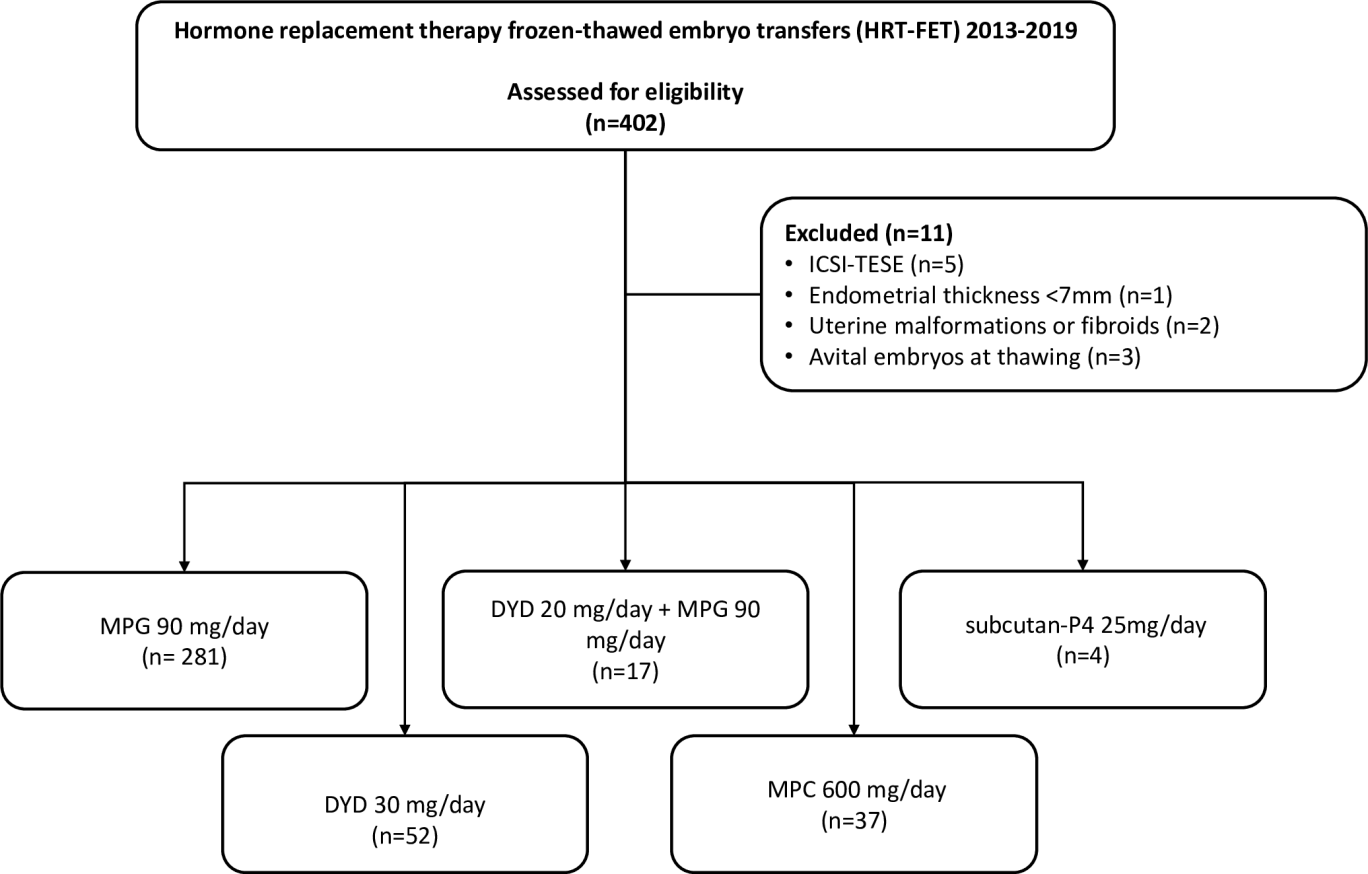
Study flow chart.

A total 391 out of 402 HRT-FET cycles were included (**Figure 1**). The number of participants per group was distributed as follows: MPG (n = 281), DYD (n = 52), DYD+MPG (n = 17), MPC (n = 37), and subcutan-P4 (n = 4).

### Baseline characteristics

The demographic characteristics are shown in **Table 1**. The patients were between 26 and 46 years of age; the median age was 35 years (interquartile range IQR 32-38 years). Regarding baseline characteristics, except for ages the study groups did not present remarkable differences e. g. sterility type (primary, secondary) (p =0.096), fertilization methods (IVF vs ICSI) (p =0.43) and number of previous transfers (0, 1 and >2) (p=0.095). Women in the Subcutan-P4 group were significantly older. The most frequent reason for infertility was anovulation/dysovulation/polycystic ovary syndrome (71% in the MPG group, 56% in the other groups). Less pronounced differences were found among the study groups regarding the type of infertility, the fertilization technique (IVF vs ICSI), and the number of previous transfers (0, 1, or > 2) (**Table 1**).

### Cycle characteristics

We included the cleavage stage embryos as well as the blastocyst stage embryos. Blastocyst stage embryo, it was possible to include as of 2017, due to modifications of the Swiss law and the possibility of blastocyst culture and preference of single embryo transfer (FMedG) (17).

The proportions of blastocysts per transfer were 56/281 (20%) for MPG, 41/52 (79%) for DYD, 14/17 (82%) for DYD+MPG, 32/37 (86%) for MPC, and 4/4 (100%) for Subcutan-P4 (p < 0.001). The proportion of single transferred embryos was higher for the DYD (85%), DYD+MPG (71%), MPC (89%), and subcutan-P4 (75%) groups as compared to the reference group MPG (35%, p < 0.001) (**Table 1**).

### CPR (Primary outcome) and LBR

The primary outcome results for clinical pregnancy showed a higher CPR in the oral DYD group (59%, 95% CI (39%-82%)) and in the combined oral DYD+MPG group (42%, 95% CI (29%-57%)), as compared to the reference group MPG (18%, 95% CI (14%-23%)) and the MPC group ((8%, 95% CI (2%-22%)).

The LBR was higher in the oral DYD group (27%, 95% CI (16%-41%)) and in the combined oral DYD+MPG group (29%, 95% CI (10%-56%)) than in the MPG group (12%, 95% CI (8%-16%)) and the MPC group (5%, 95% CI (1%-18%)). In the Subcutan-P4 group, one out of four transfers (25%) led to clinical pregnancy and live birth (**Table 2**).

**Figure 2:**
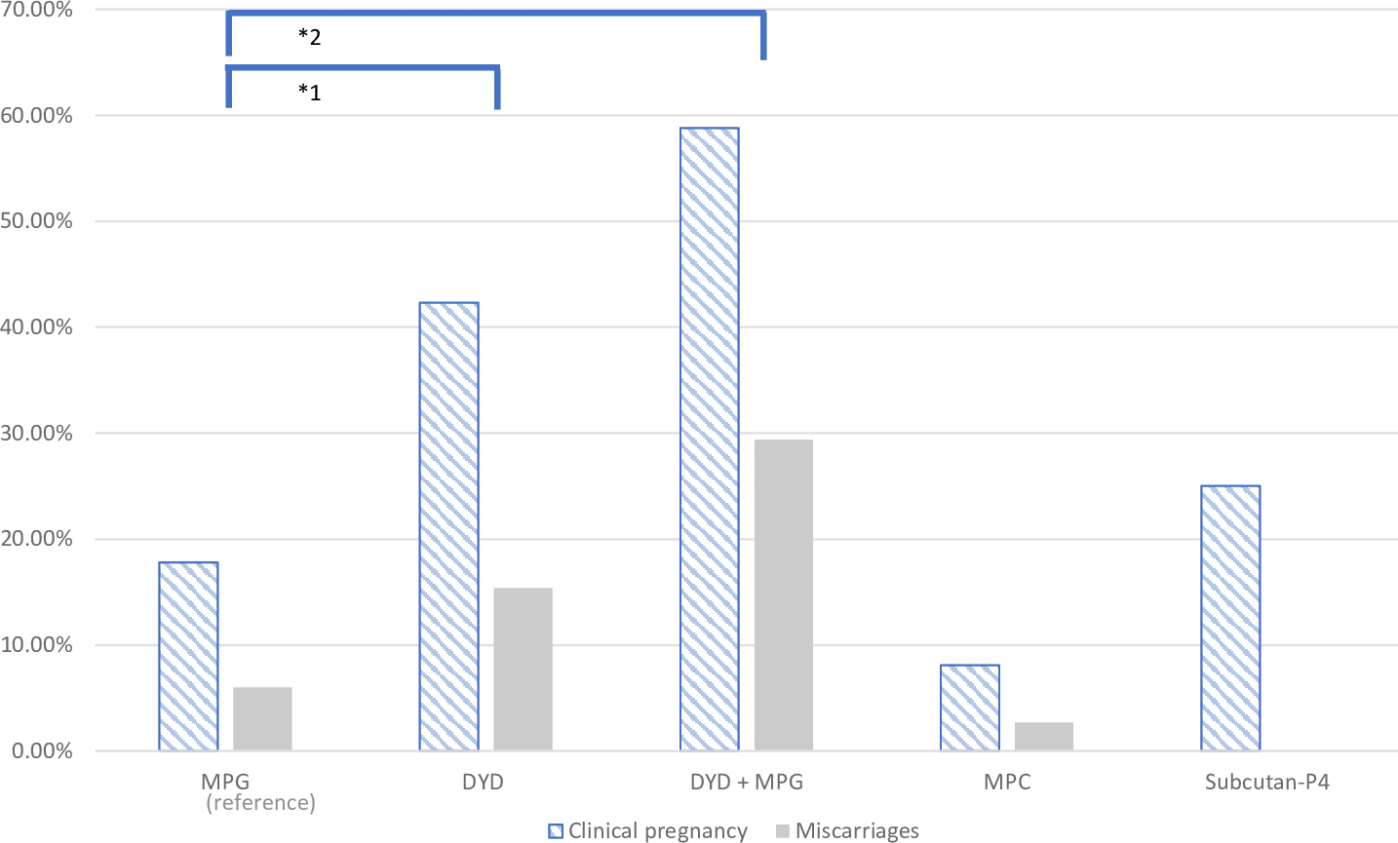
Graphical representation of clinical pregnancy and miscarriage rate in the five progesterone groups.

The unadjusted ORs for the clinical pregnancy rates, using MPG as a reference, were OR = 3.39 (95% CI 1.81-6.36, p < 0. 001) in the oral DYD group and OR = 6.60, (95% CI 2.40-18.18, p < 0.001) in the combined oral DYD+MPG group. With regard to LBRs, the unadjusted analysis showed an OR = 2.77 (95% CI 1.36-5.64, p = 0.005) for the DYD group and OR = 3.13 (95% CI 1.04-9.45, p = 0.043) for the DYD+MPG group (**Figure 2** and **Table 3**).

**Table 3:** Primary outcomes: multiple logistic regression analyses of clinical pregnancy and live birth rate.

	Outcome: Clinical pregnancy							
	Unadjusted				Adjusted			
	OR	95% CI unten	95% CI oben	P-Value	OR	95% CI unten	95% CI oben	P-Value
Treatment group								
*(Reference: MPG)*								
DYD	**3.39**	**1.81**	**6.36**	**<0.001**	**2.87**	**1.38**	**6.00**	**0.005**
DYD + MPG	**6.60**	**2.40**	**18.18**	**<0.001**	**5.19**	**1.76**	**15.36**	**0.003**
MPC	0.41	0.12	1.38	0.15	0.36	0.10	1.34	0.13
Subcutan-P4	1.54	0.16	15.11	0.71	1.35	0.12	14.92	0.81
	Outcome: Live birth							
	Unadjusted				Adjusted			
	OR	95% CI unten	95% CI oben	P-Value	OR	95% CI unten	95% CI oben	P-Value
Treatment group								
*(Reference: MPG)*								
DYD	**2.77**	**1.36**	**5.64**	**0.005**	**2.58**	**1.11**	**6.00**	**0.028**
DYD + MPG	**3.13**	**1.04**	**9.45**	**0.043**	2.49	0.74	8.38	0.14
MPC	0.43	0.10	1.87	0.26	0.45	0.09	2.15	0.32
Subcutan-P4	2.51	0.25	24.79	0.43	2.02	0.17	24.56	0.58

Unadjusted and adjusted analysis for age, type of sterility, cleavage-stage embryo vs blastocyst, and the number of the transferred embryo.

Oral dydrogesterone 30 mg/day (DYD), vaginal progesterone gel 90 mg/day (MPG), a combination of DYD 20 mg/day and MPG 90 mg/day (DYD+MPG), vaginal progesterone 600 mg (MPC) and subcutaneous injection of 25 mg/day progesterone (subcutan-P4).

In the multiple logistic regression analysis, after adjusting for the predefined potential confounders mentioned above, the ORs were similar but slightly less pronounced. A increase in clinical pregnancy was observed in the oral DYD group (OR = 2.87, 95% CI 1.38-6.00, p = 0.005), and in the combined DYD+MPG oral group (OR = 5.19, 95% CI 1.76-15.36, p = 0.003), compared to MPG as the reference group. In terms of live births, the rate in the oral DYD was again higher than in the MPG group, with an OR = 2.58 (95% CI 1.11-6.00, p = 0.028) and showed a similar trend for the DYD+MPG group, with an OR = 2.49 (95% CI 0.74-8.38, p = 0.14).

Numbers in bold represent statistically significant.

In the multiple logistic regression analysis, after adjusting for the predefined potential confounders mentioned above, the ORs were similar but slightly less pronounced. A increase in clinical pregnancy was observed in the oral DYD group (OR = 2.87, 95% CI 1.38-6.00, p = 0.005), and in the combined DYD+MPG oral group (OR = 5.19, 95% CI 1.76-15.36, p = 0.003), compared to MPG as the reference group. In terms of live births, the rate in the oral DYD was again higher than in the MPG group, with an OR = 2.58 (95% CI 1.11-6.00, p = 0.028) and showed a similar trend for the DYD+MPG group, with an OR = 2.49 (95% CI 0.74-8.38, p = 0.14) (**Table 3**).

Based on several sensitivity analyses, the estimated treatment effects and ORs for luteal treatment were found to be quite robust, even after accounting for additional covariates such as grade of infertility, reason for infertility, method of fertilization, number of previous transfers, and calendar year of transfer. Essentially, none of the covariates (including the pre-specified covariates) demonstrated a stronger effect than treatment supporting the luteal phase. Stepwise model selection using Akaike’s or Bayesian information criteria even led to the proposition that a model with luteal treatment as the only independent variable – for explaining rates of clinical pregnancies or live births – was preferable to models with predefined or additional covariates. However, it should also be noted that none of the models achieved a Pseudo R^2^ greater than 11%, suggesting that none of the models could truly achieve high predictive power and that the true chance of clinical pregnancies or live births remains difficult to explain.

### Secondary outcomes

No notable differences were observed among the five groups (DYD, MPG, DYD+MPG, MPC, and subcutan-P4) in the rates of biochemical pregnancy only (five events in total, three of which occurred in the MPG group, p=0.51) or the ectopic pregnancy (one event in total, which occurred in the MPG group, p=1.0). Vaginal bleeding in early pregnancy and obstetric complications were presented in the following progesterone groups: DYD, DYD+MPG, MPC, and subcutan-P4, i.e., all except for the MPG group. There were no complications during late pregnancy reported.

In terms of miscarriages rate, the DYD+MPG group was the highest, with 29%, followed by 15% for DYD and 7% for MPG (p=0.005). **(** **Figure 2** **).** In the multiple logistic regression analysis after adjusting, shows an increased rate of miscarriage in the oral DYD group (OR = 2.94, 95% CI 0.76-11, p = 0.12), and in the combined DYD+MPG oral group (OR =6.02, 95% CI 1.45-124.91, p = 0.013), compared to MPG as the reference group. No difference is observed in ET blastocyste vs cleveage stage and Year of ET.

## Discussion

The present study was designed to compare the clinical reproductive outcomes among five types of progesterone applications (DYD, MPG, DYD+MPG, MPC, and subcutaneous-P4) in real-world FET cycles. The LBR was higher in the oral DYD alone group and in the DYD + MPG group, compared with the MPG group. DYD and the combination of oral DYD+MPG showed a higher CPR compared with MPG.

Even though there are no guidelines, vaginal progesterone is the most common route for luteal phase support in Europe (17) and at our center as well. We consequently selected MPG as the reference group for our study. Intramuscular and subcutaneous applications are effective; however, these routes are not preferred due to the difficulty of application by the patient and the potential side effects (17–19). Comparing intramuscular progesterone and MPC, and the combination of the two, in an American randomized controlled trial (RCT) showed that vaginal-only progesterone replacement in FET was associated with a decrease in ongoing pregnancy, due to increased miscarriages compared with intramuscular application or the progesterone combination (20). In addition, it also shows that the combination of vaginal progesterone supplemented with intramuscular progesterone every three days was not inferior to daily intramuscular progesterone. More studies are needed to validate this finding.

DYD is a synthetic progestin with high oral bioavailability and selectivity for P4 receptors; it is therefore suggested as an alternative to MPG and MPC in LPS (21, 22). Two large-scale phase III RCTs (the LOTUS I and LOTUS II studies (n ≥ 2,000)) concluded that there were no differences in pregnancy rates at 12 weeks’ gestation and in the LBR when comparing the use of DYD and micronized vaginal progesterone (14, 15) in fresh ART cycles. Oral DYD is proposed as an LPS option because of good tolerability, a patient-friendly route of administration, and high efficacy. In 2020, a systematic review and meta-analysis concluded that oral DYD was associated with a higher pregnancy rate and LBR than micronized progesterone administered in a capsule or vaginal gel in fresh ART cycles (13).

In terms of FET cycles and LPS, we identified three representative studies comparing DYD with other types of progesterone supplementation in the luteal phase. In the first study, Rashidi et al. (2016) compared oral DYD and intramuscular support. Their results showed similar CPR and LBR as with DYD and intramuscular P4; therefore, they suggest that using DYD in FET cycles should be implemented due to its ease of use, lower cost, and higher patient satisfaction (23). A large (N = 1364) prospective Vietnamese cohort study compared DYD+MPG vs. MPG in HRT-FET cycles. Oral DYD + MPG showed a higher live birth rate (RR 1.30 (95% CI 1.01–1.68), P=0.042) and a lower rate of miscarriages (MPG) vs. (DYD + MPG) (3.4% versus 6.6%; RR 0.51, 95% CI 0.32–0.83; P=0.009) (24). Our study also shows a higher CPR under LPS treatment with DYD and DYP+MPG.

In the third study, an Iranian RCT, the authors compared four LPS regimens including MPC 400 mg/d, DYD 20 mg/d, a combination of DYD 20 mg/d and gonadotropin-releasing hormone analog, and a combination of DYD 20 mg/d and human chorionic gonadotropin. Their results showed that the DYD-only group had a lower CPR than the other three groups (25). There were no significant differences among the four groups in terms of the ongoing pregnancy rate or miscarriage rate. The authors’ hypothesis regarding the low pregnancy rate when using DYD alone was the reduced dosage of DYD of 20 mg/d compared to a dosage of 30 mg/d.

In our study, we used oral DYD doses three times a day, for a total of 30 mg. There were differences in the administration in various protocols of clinical studies on fresh-cycle IVF and HRT-FET of DYD: three times daily vs. twice daily (25–30), with our approach favoring three times a day. As for the intramuscular dose of progesterone, we also find different references for doses ranging from 25 to 200 mg/day (31, 32). The current literature review shows that the dose of 25 mg/day offers good prospects for efficacy in supporting the luteal phase (31, 32).

In terms of secondary Outcomes, In our study, a higher number of miscarriages was observed in the DYD + MPG group (29%) compared with the reference group (p=0.019). Double administration of progesterone was preferred by patients with recurrent implantation failure, which may explain the high number of miscarriages in this group. In terms of the current literature, Devall, et al., 2021, in a network meta-analysis, demonstrated that vaginal micronized progesterone can increase effectively the LBR in women with a history of recurrent miscarriages and bleeding in early pregnancy. Concerning the comparison of vaginal progesterone and intramuscular progesterone was observed in a RCT that 50% of pregnancies of women who received only vaginal progesterone ended in miscarriage, although the combination of vaginal and intramuscular progesterone (every 3 days) could also be an effective regimen (20).

A lingering question about the use of DYD is the maternal and fetal safety of the drug, given that DYD is a synthetic progesterone and is therefore not identical to ovarian P4. Our study observed no increased incidence of maternal or fetal complications. A retrospective study by Zaquot et al. (2015) suggested a low rate of congenital heart disease in infants born to mothers exposed to oral DYD in the first trimester of pregnancy (33). This hypothesis has not been verified in clinical studies; however, prospective studies on the teratogenic risk of oral DYD provide a strong view on its safe use in LPS (14, 24, 30).

### Application

The use of oral DYD by women in our study was well tolerated. The oral administration is more convenient for patients than intravaginal administration due to the association of micronized vaginal progesterone with irritation and increased vaginal discharge (15, 34). Recently, it has been postulated that the endometrium is resistant to progesterone due to alterations in the uterine microbiome through vaginal progesterone application (13, 35). In addition, a clinical study suggested that micronized vaginal progesterone affects the composition of the vaginal and endometrial microbiota (24). These findings indicate that the use of vaginal progesterone could contribute to an unbalanced microbiome of the female upper genital tract, resulting in reduced progesterone absorption in the vagina and, consequently, decreased P4 levels in the uterus. This can in turn lead to a decreased implantation rate and an increased miscarriage rate (24).

### Strengths and limitations

The CPR is used in our study as main outcome in fertility treatment; this is the appropriate parameter assessed in the setting of a clinical trial (36). Another strength is that the estimated treatment effects and ORs for luteal treatment were found to be robust and provided similar estimates from unadjusted and adjusted logistic regressions, including sensitivity analyses. The estimated treatment effects and ORs for luteal treatment were found to be robust, even after accounting for additional covariates such as type of infertility, reason for infertility, method of fertilization, number of previous transfers, and calendar year of transfer and development of embryo. Essentially, none of the covariates (including the pre-specified covariates) demonstrated a stronger effect than progesterone treatment.

Various limitations of our study should be considered. First, it was a single-center, retrospective study with no randomization of the LPS. However, the real-world population of all 392 FET cycles performed at our clinic meeting inclusion criteria included is an advantage, because it represents fully every day clinical reality. Second, since Swiss law was modified in 2017, assisted reproductive medicine procedures and techniques have further evolved. In our study, we present the results from 2013 to 2019 in HRT-FET, in which embryos transferred were at the cleavage and blastocyst stages with an overrepresentation of blastocyst stage transfers in the reference group (MPG); however, a corresponding adjustment was made to our statistical method. Several high-ranked studies mentioned above (14, 15) included blastocyst and cleavage stage embryo transfers within the same sample.

## Conclusions

In conclusion, the literature shows that DYD is well-tolerated and probably contributes to the immunomodulation of the receptive endometrium. It can therefore be applied for luteal phase support in FET cycles. In this study, the addition of DYD in luteal phase support in artificial frozen-thawed embryo transfer cycles was associated with higher CPR and LBR than the use of MPG alone.

## Data availability statement

The original contributions presented in the study are included in the article/supplementary material. Further inquiries can be directed to the corresponding author.

## Ethics statement

The studies involving human participants were reviewed and approved by the Business Administration System for Ethics Committees (BASEC 2020-01527). The patients/participants provided their written informed consent to participate in this study.

## Author contributions

AV and CD contributed to the design of study. AV performed studies search and data collection. DL performed statistical analyses and interpretation of data. Write, Review and Editing: AV, CD, NW, JW, DL, AS. All authors contributed to the article and approved the submitted version.
